# Ketamine effects on EEG and their links to therapy differ across treatment-resistant major depression, post-traumatic stress disorder, and obsessive-compulsive disorder

**DOI:** 10.1093/ijnp/pyag037

**Published:** 2026-07-06

**Authors:** Shabah M Shadli, Neda Nasrollahi, Calvin K Young, Gabrielle S R Schuck, Meadow G Whatson, Tame N J Kawe, Shona Neehoff, Ben Beaglehole, Paul Glue, Neil McNaughton

**Affiliations:** Department of Psychology, University of Otago, Dunedin, New Zealand; Department of Psychology, Charles Sturt University, Bathurst, NSW, Australia; Department of Psychology, University of Otago, Dunedin, New Zealand; Department of Psychology, University of Otago, Dunedin, New Zealand; Department of Psychology, University of Otago, Dunedin, New Zealand; Department of Psychology, University of Otago, Dunedin, New Zealand; Department of Psychology, University of Otago, Dunedin, New Zealand; Department of Psychology, University of Otago, Dunedin, New Zealand; Department of Psychological Medicine, University of Otago, Christchurch, New Zealand; Department of Psychology, University of Otago, Dunedin, New Zealand; Department of Psychology, University of Otago, Dunedin, New Zealand

**Keywords:** ketamine, depression, post-traumatic stress disorder, obsessive-compulsive disorder, EEG

## Abstract

**Introduction:**

Neurotic disorders—major depressive disorder (MDD), panic disorder, social anxiety disorder, generalized anxiety disorder, obsessive-compulsive disorder (OCD), post-traumatic stress disorder (PTSD), and specific phobia—have differing pharmaceutical profiles. But all, even when resistant to conventional treatment (TR), respond quickly to low-dose (0.5-1.0 mg/kg I.M.) ketamine. We explore the variation in the neural effects of ketamine across its treatments of TR-MDD, TR-PTSD, and TR-OCD.

**Materials and methods:**

We recorded 10 min of resting frontal activity and diagnosis-related scale measures, before and 2 h after fentanyl (50 mcg) or ketamine (0.5 or 1.0 mg/kg, I.M.) counterbalanced across 3 sessions at least a week apart. Average power spectra were calculated for delta, theta, alpha1, alpha2, beta, and gamma bands. Analysis of variance compared TR-PTSD (20 F, 2 M) with TR-MDD (12 F, 13 M). Preliminary TR-OCD (5 F, 2 M) data were also obtained.

**Results:**

TR-PTSD patients showed dose- and band frequency-dependent electroencephalography (EEG) power changes (particularly alpha at 0.05 mg/kg), while TR-MDD patients did not. TR-OCD differed qualitatively from both. The correlation of power change with score change was maximal for different bands and electrodes across the different scales (Impact of Events Scale-Revised, Montgomery-Åsberg Depression Rating Scale, Hospital Anxiety and Depression Scales, Hamilton Anxiety Scale, Fear Questionnaire, and Yale-Brown Obsessive-Compulsive Scale).

**Discussion:**

Ketamine effects and their therapeutic links vary in band and site with conventional diagnosis—including previous TR anxiety results. The EEG results appear to detect changes in the disorder-specific systems that conventional treatments target selectively and directly and these appear to require a ketamine-sensitive factor (as a “double hit”) to generate disorder.

## Introduction

There has always been a tension in psychiatric diagnosis between “lumping” and “splitting”. Here we offer data that suggest these positions are not incompatible.

In the past, “anxiety disorders” were split into “six main types (panic disorder, social anxiety disorder, generalized anxiety disorder, obsessive-compulsive disorder, post-traumatic stress disorder, and specific phobia) each of which has characteristic symptoms and cognitions.”^1^ Now, obsessive-compulsive disorder (OCD) and post-traumatic stress disorder (PTSD) are seen as distinct from anxiety,^2^^,^^3^ as is major depressive disorder (MDD). Most of these diagnoses differ in their selective lack of response to some classes of conventional pharmacological treatments, consistent with a splitting perspective (see Table 1).^4^ However, they are all responsive to Specific Serotonin Reuptake Inhibitors (SSRIs), which would be consistent with a lumping perspective.

**Table 1:** Demographic data—mean (SD).

	PTSD	MDD	OCD
**Age, year**	32.3 (9.8)	32.4 (11.4)	33.4 (9.7)
**Weight, kg**	82.0 (24.1)	79.6 (18.5)	80.3 (17.8)
**Gender**	16f, 6m	12f, 13m	7f, 3 m
**Baseline IESR**	50.3 (16.0)	-	-
**Baseline HADS-A**	-	13.2 (4.6)	-
**Baseline HADS-D**	-	13.6 (3.7)	-
**Baseline YBOCS**	-	-	29.9 (3.9)
**NFA**	3.6 (1.7)	3.5 (1.6)	3.9 (1.7)

Abbreviations: NFA, Number of failed antidepressants prior to study entry; PTSD, post-traumatic stress disorder; MDD, major depressive disorder; OCD, obsessive-compulsive disorder.

In terms of lumping, it has been argued that all of these disorders can be grouped together within a general neurotic syndrome.^5^ They also all have cases that are “treatment-resistant” (TR), failure to respond to at least 2 conventional drugs and psychotherapy.^6^ Here, the “neurotic” label is consistent with a common effectiveness of treatment with ketamine in TR cases of MDD^7^^,^^8^:, PTSD^9^^,^^10^: OCD^11^^,^^12^. For general reviews, see references.^13^^,^^14^

Ketamine is anesthetic, analgesic, and dissociative. These effects are related to different doses with efficacy in TR-MDD limited to a particularly narrow low-dose band.^15^ Its mechanisms of action are unclear but complicated. It is often classified as “an NMDA receptor antagonist” (which would tend to block neural plasticity). But its key action may be at metabotropic, not ionotropic, glutamate receptors. But other metabotropic NMDA antagonists (which do not cause a release of glutamate) are not so effective.^16^ The key effects of ketamine appear to involve increased plasticity.^17^ Its primary therapeutic action on brain processes is unknown.

**Figure 1:**
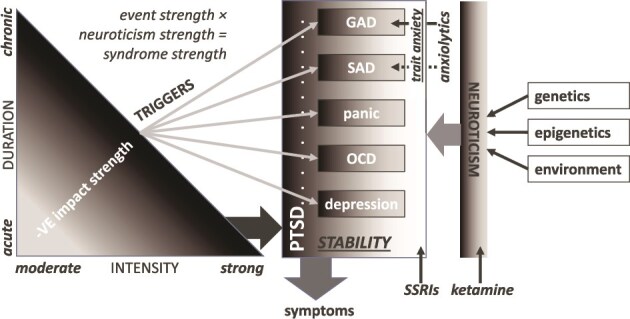
Diagrammatic representation of the double-hit hypothesis for disorders known to be affected by ketamine. Disorder is held to result when high neuroticism (resulting from genetic, epigenetic, and prior environmental factors) is combined with a high level of a disorder-specific trait linked to GAD, SAD, panic, OCD, or depression. High levels of these specific traits can be triggered by moderate chronic or strong acute stress. In the latter case, PTSD may result. Specific anxiolytic drugs, ie, those with anxioloytic but not panicolytic action, affect the specific personality trait linked to GAD and to a lesser extent SAD. SSRIs act on a background higher order trait of “stability,”^28^ which would not be specific to aversive events^29^ and which would slowly alter the specific trait levels. Ketamine acts to affect neuroticism and so alters the disordered expression of all the specific traits. Figure recolored and legend adapted from^4^ under the terms of the Creative Commons Attribution License (http://creativecommons.org/licenses/by/4.0/)

EEG can monitor multiple brain processes in parallel. Specific tasks can be used to test specific theoretically predicted changes. But for more general comparative work, it is common to use resting (eyes open alternating with eyes closed) EEG, eg.^18–25^ We linked the therapeutic action of ketamine on generalized and social anxiety (GAD/SAD) to resting-state drug-induced right-frontal changes in the “theta” frequency band.^26^ Right-frontal theta is sensitive to selective anxiolytic drugs that do not affect depression, panic, or obsession.^27^ Ketamine, then, may be acting on a general “neurotic” system to produce the same ultimate changes in neural activity as a disorder-specific conventional treatment (Figure 1)—with the disorder requiring a “double-hit” combining neuroticism with a disorder-specific trait.^4^ So, here, we use the same procedures as Shadli, Kawe, Martin, McNaughton, Neehoff, and Glue^26^ to explore changes in the EEG during ketamine therapy of MDD, PTSD, and OCD. Our exploratory question was: does ketamine produce disorder-specific or disorder-general effects?

## Methods

### Details reported previously for safety and efficacy studies in the same patients

We analyzed EEG data collected in patients treated with ketamine and for whom we have previously published safety and efficacy data in Glue, Neehoff, Beaglehole, Shadli, McNaughton, and Hughes-Medlicott^7^ for MDD, Beaglehole, Glue, Neehoff, Shadli, McNaughton, Kimber, Muirhead, de Bie, Day, and Hughes-Medlicott^9^ for PTSD, and Beaglehole, Glue, Neehoff, Shadli, McNaughton, Kimber, Muirhead, de Bie, Day-Brown, and Hughes-Medlicott^11^ for OCD. Full methodological and safety details are in the Supplementary online material. This was a 3-way within-subject double-blind active-controlled cross-over design in patients with TR-MDD or TR-PTSD DSM-5^2^ and involved a direct comparison of the two diagnoses. We also report preliminary significant results for TR-OCD in a small sample (limited by recruitment problems and data loss) for further comparison. A CONSORT diagram is provided as a graphical abstract.

Single doses of racemic ketamine 0.5 mg/kg, 1.0 mg/kg, or fentanyl 50 mcg (psychoactive control) were administered as intramuscular injections in the deltoid muscle—with balanced randomization (but see OCD variation in Supplementary Methods). Three dosing sessions were each separated by at least 1 week. A 10-min relaxation EEG test was obtained pre-dose and 2 and 24 h after each dosing session. Note that the half-life of fentanyl is longer than that of ketamine, but, as a result of its different pharmacology, it is not therapeutically effective at the 20-h time point focused on here.

The authors assert that all procedures contributing to this work comply with the ethical standards of the relevant national and institutional committees on human experimentation and with the Helsinki Declaration of 1975, as revised in 2008. See supplementary methods for details.

### Participants for EEG testing

#### Initial baseline demographics

Demographics are provided in Table 1. All patients continued on their existing medication (Table 2).

**Table 2:** Current medication (percent of patients).

Drug class	OCD	PTSD	MDD
**Aripiprazole**			5%
**Bupropion**		7.7%	15%
**Lamotrigine**		7.7%	
**Mirtazapine**			5%
**None**		7.7%	25%
**Nortriptyline**		7.7%	
**Quetiapine**	16.7%	30.8%	5%
**SSRI**	50%	53.8%	40%
**Venlafaxine**	33.3%	23.1%	20%

Abbreviations: MDD, major depressive disorder; OCD, obsessive-compulsive disorder; PTSD, post-traumatic stress disorder.

#### MDD and PTSD

The original complete pool of participants for safety and efficacy was 25 TR-MDD (50 screened, 29 enrolled, and 4 withdrawn) and 33 TR-PTSD (78 screened, 34 enrolled, and 1 withdrawn). Due to the unavailability of an EEG operator (due to, eg, COVID), the initial 58 was reduced for the current experiments to 47 patients in total, where 22 received a primary diagnosis of PTSD and 25 MDD. In the PTSD cohort, there were 20 females and 2 males aged 20-50 years. Of these, 3 patients had a secondary diagnosis of GAD/SAD, 1 of OCD, and 13 of MDD. These comorbid cases were included in the PTSD group in the initial analysis comparing the diagnoses but then excluded from a post-hoc analysis focusing on PTSD. In the TR-MDD cohort, there were 12 females and 13 males in the TR-MDD group aged between 19 and 54 years. Of these, 6 were comorbid with GAD/SAD but not PTSD. There were 11 comorbid with PTSD. These PTSD cases were included in the MDD group in the initial analysis comparing the diagnoses but then excluded from a post-hoc analysis focusing on MDD.

#### OCD (preliminary)

The original complete pool of TR-OCD participants for safety and efficacy was 10 (24 screened, 12 enrolled, and 2 withdrawn). Of the 10, 3 were excluded from analysis due to missing key session data, leaving 2 males and 5 females for analysis. Given recruitment problems for any additional work and statistical significance in the analyses, we are reporting the results as preliminary here to allow comparison with MDD and PTSD.

The patients were clinically assessed at 0, 1, 2, 24, 72, and 168 h relative to drug dosing for safety and efficacy testing as reported previously.^7^^,^^9^^,^^11^ EEG was acquired at 0, 2, and 24 h timepoints relative to dosing. We report only EEG data obtained at 0 (pre) and 2-h (post) dosing timepoints in the current study to determine the direct effects of ketamine given its short half-life.

### E‌EG recording

EEG was acquired via a Waveguard EEG cap (ANT Neurotechnology) in the 10:20 configuration to cover Fp1, Fp2, F7, F3, Fz, F4, F8, C3, Cz, C4, P7, P3, Pz, P4, P8, M1, and M2 electrode positions. The recording reference was M1 + M2 (connected together electrically). Cap size was determined by patients’ head circumference, with 3 available sizes: large (57-64 cm), medium (53-57 cm), and small (47-53 cm). Electrode gel was applied to reduce and normalize the impedance across the recording electrodes (<10 kΩ, with Pre-Check Plus). The EEG cap was connected to 2 Bioradio devices (Cleveland Medical Devices Inc.), which streamed data via Bluetooth at a sampling rate of 256 Hz to a computer using BioCapture software (Cleveland Medical Devices Inc.) for subsequent offline analysis.

Patients were fitted with the EEG cap prior to a pre-dose recording session. They were instructed to sit still to minimize movement artifacts and alternated between keeping their eyes open and closed for 1 min intervals over a 10 min period. Markers in the EEG file indicated the start and end of the alternation. Patients then received intramuscular administration of 50 mcg fentanyl (active control), and 0.5 or 1 mg/kg of ketamine in counterbalanced order across 3 different weeks. At 2 h post-dose, EEG recording of 10 min of relaxation was repeated.

### Clinical ratings analysis

To link EEG changes to changes in mental state, we used changes in clinical scores between pre-dose recording and 2-h post-dosing recording (ie, post-pre) as a surrogate measure for improvement. The primary clinical rating used for PTSD is the Impact of Event Scale-Revised (IES-R, *n* = 22). The Montgomery-Åsberg Depression Rating Scale (MADRS), Hospital Anxiety and Depression Scale (HADS-A and HADS-D) were used for MDD (*n* = 25) and the Yale-Brown Obsessive-Compulsive Scale (Y-BOCS) for OCD (*n* = 7). There were 11 MDD patients with a secondary diagnosis of PTSD who also received IES-R assessment. For patients who also suffer from anxiety disorders as a secondary diagnosis (*n* = 14, *n* = 2 from the PTSD cohort), HAM and FQ were also assessed between drug dosing. Relevant clinical ratings for comparison with EEG changes (IES-R, MADRS, HADS, HAM, FQ, and Y-BOCS) were obtained at pre-dosing and 2 h after dosing for each dose, delivering 3 different values per participant. For each participant, these values were centered (ie, their average for that participant was subtracted) to eliminate any subject-wise variance, leaving a dose-related residual, which was used for the correlation analyses. For correlations, therefore, n were PTSD (66), MDD (75 but with some scores missing for some scales), and OCD (21).

### E‌EG analysis

#### Pre-processing

The raw EEG data were imported into MATLAB for analysis, with most scripts taken from EEGLAB.^30^ Participants with incomplete recording sessions, corrupt files, and bad EEG (flat channels, noise with no signal) were excluded (see data summary). First, the data were high-pass (1 Hz) filtered and then low-pass filtered (40 Hz) using a zero-phase digital filter (*filtfilt()* function in MATLAB). The recordings from the 2 devices were then synchronized by using the lag with the highest cross-correlation coefficient between the averaged signals from each recording. Once aligned, we performed Independent Components Analysis (ICA) on the combined recordings using *runica()* with a learning rate of 0.001 at 200 maximum iterations. Mains noise was removed using *cleanline()* with default parameters except for “*Bandwidth,*” which was set at “2.” Subsequently, *clean_artifacts()* was used to remove slow drifts, flat-line channels, movement artifacts, and other potential noise with default parameters; heavily affected channels were interpolated using *pop_interp()* with spherical interpolation using channel location data. Once cleaned, the EEG was then referenced by using REST, a re-referencing technique to derive a theoretical electrically neutral reference using source modeling,^31^ and re-sampled at 128 Hz for spectral analysis.

#### Spectral analysis

The time-frequency multi-taper spectral analysis was carried out using Chronux.^32^ A 2 s window with 95% overlap (100 ms) was used with 3 tapers and a numerical bandwidth of 5. Frequencies between 0 and 70 Hz were analyzed. The band-specific spectral power was log-transformed and averaged at their respective ranges: delta (1-3 Hz), theta (4-6 Hz), alpha1 (7-9 Hz), alpha2 (10-12 Hz), beta (25-34 Hz), and gamma (41-53 Hz).

#### Higuchi fractal dimension (HFD)

The complexity of EEG was assessed using Higuchi’s method^33^ with *k*_max_ = 8. After pre-processing, the data were band-pass filtered between 2 and 36 Hz. Similar to the spectral analysis described below, a 2 s analysis window was used but at 50% overlap. The cumulative sample-to-sample differences in each window as per *k* yields a slope where a low value indicates a more proportional signal, hence less complexity and vice-versa for a high HFD value.

#### Alpha asymmetry

The log spectral power from 2 alpha bands (7-9 Hz and 10-12 Hz) was subtracted at F8-F7 and F4-F3 pairings. This yielded 4 right–left alpha asymmetry values.

#### Post-processing

To facilitate a focused analysis of EEG changes in the rostro-caudal direction, Fp1 and Fp2 were averaged post-referencing to derive a surrogate “Fpz” to create an Fpz-Fz-Cz axis.

#### Data summary

The final EEG dataset after exclusions during preprocessing included 12 (11/1 female/male) from the PTSD cohort and 21 (10/11 female/male) from the MDD cohort. Although all recording channels were used for referencing with REST, data from only frontal channels (Fp1, Fp2, F7, F3, Fz, F4, F8, and Cz) are reported here for consistency with Shadli, Kawe, Martin, McNaughton, Neehoff, and Glue.^26^

### Statistics

#### Analysis of variance

All statistics were performed using the repeated measures analysis of variance (ANOVA) in SPSS. SPSS routinely extracted orthogonal polynomials for channel and frequency band. Note that these polynomial components are independent, additive, and purely descriptive - see Figure 1 in reference.^34^ There is no assumption of any specific underlying mathematical function. As their contrasts have 1 degree of freedom and distinct error terms, they avoid repeated-measures problems such as sphericity. To allow this polynomial analysis for channels, electrodes were separated into 2 sets. “Left–right” was F7, F3, Fz, F4, and F8 (in that order) as levels of the channel factor. “Anterior–posterior” was Fpz, Fz, and Cz (in that order). These sets are graphed separately in the figures (with Fz repeated).

#### Correlations

Derivation of the values to be correlated is described above (clinical ratings analysis). Pearson’s *r* was used to determine correlations. Raw *P*-values are reported unless otherwise stated. Multiple comparisons were corrected with false discovery rate (FDR)-adjusted *P*-values calculated using the standard Benjamini–Hochberg procedure^35^ over the entire pool of 252 correlations.

## Results

### Changes in EEG frequency band power in MDD and PTSD

**Figure 2:**
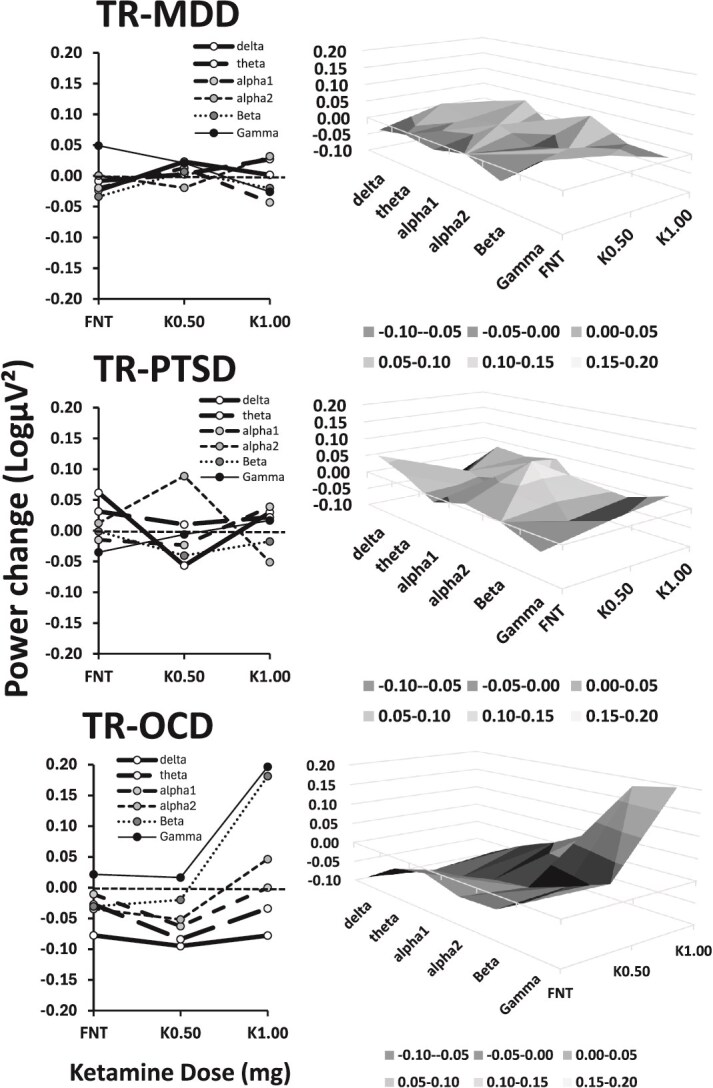
Variation in post-pre power change across treatment-resistant diagnoses by frequency band and ketamine dose, averaged across channel. Experiment 1 (including comorbid cases): major depression = MDD, post-traumatic stress disorder = PTSD. Experiment 2: (obsessive-compulsive disorder = OCD). The apparent increases in beta and gamma power are not significant; if real are likely to be due primarily to left and right frontal sites (F7, F4, F8), and are lower (Fz) or absent (Fpz, Cz) along the midline (see Figure 4)

**Figure 3:**
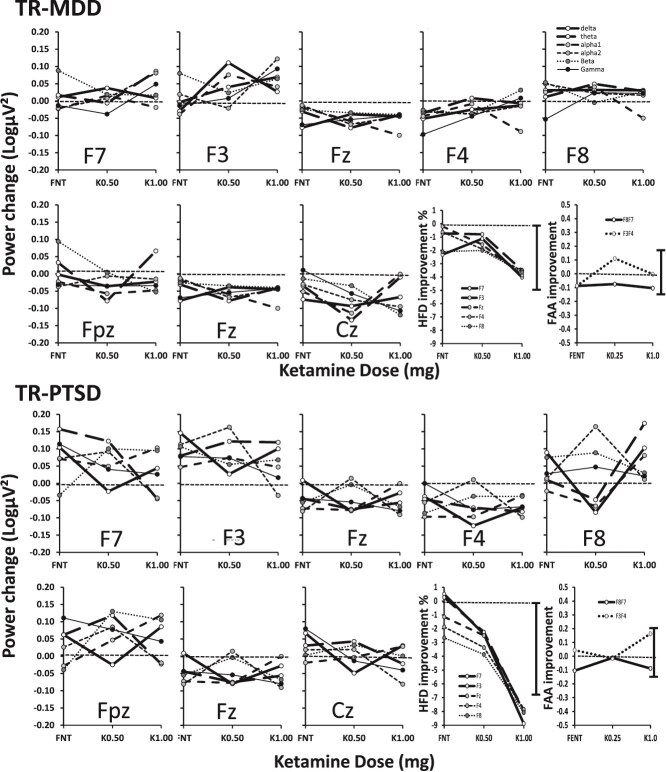
Upper and left panels: Variation in post-pre power change across treatment-resistant diagnoses (major depression = MDD, post-traumatic stress disorder = PTSD) by frequency band, channel, and ketamine dose. Bottom right panels: Improvements in Higuchi’s fractal dimension (HFD%) across frontal channels; and frontal alpha asymmetry for F8/F7 and F4/F3 electrode pairs averaged over α1 and α2 bands. Bars are the maximum of the individual data point SEs ×2 not of trend components tested statistically

Separate analyses were carried out for left–right (F7 to F8) and anterior–posterior (Fpz–Cz) sets of channels for groups based on predominant diagnosis. TR-PTSD showed power increases with both the low and high doses and with an increase in beta and decrease in delta at the low dose and a modest steady dose-related increase in gamma. TR-MDD did not show these changes (see Figure 2 for channel average; left–right, diagnosis × dose [quadratic] × frequency [quadratic], *F*(1,31) = 7.452, *P* = .01; diagnosis × dose [linear, quadratic] × frequency [order 5], *F*(1,31) = 7.795, 11.657, *P* = .009, .002). These effects did not interact significantly with the channel (see Figure 3 for channel details; all channel effects *F*(1,31) < 3.2, *P* > .086). With 6 frequency bands, order 5 indicates that just 1 frequency band differs from the lower order trends shared with the other 5 and can be attributed to the increased alpha2 at 0.5 mg/kg (quadratic component) dropping to a decrease at 1.0 mg/kg (linear component). Given the diagnostic differences in power, we carried out separate post-hoc ANOVAs for the 2 diagnoses.

For TR-MDD, there may have been a frequency-specific difference between F7 and F8 at the highest ketamine dose (left–right, channel [linear] × dose [linear] × frequency [order 4], *F*(1,20) = 5.766, *P* = .026). There is a general decrease in power at 0.5 mg/kg for the AP channels (dose [quadratic, *F*(1,20) = 6.434, *P* = .020) and its overall lesser effect at Fz is not reliable (channel [quadratic] × dose [quadratic], *F* = 2.153, *P* = .158, NS), but there may be a similar diminution for 1.0 mg ketamine across some frequency bands (channel [quadratic] × dose [linear] × frequency [order 4], *F* = 4.847, *P* = .040).

For TR-PTSD, the overall general U-shaped effect of dose at F7 and inverted-U at F8 appeared reliable (channel [linear] × dose[quadratic], *F*(1,11) = 7.919, *P* = .017) but varied across the frequency bands (channel [linear] × dose [quadratic] × frequency [cubic], *F*(1,11) = 8.903, *P* = .012) with an increased spread at the higher dose (channel [linear] × dose[linear] × frequency[cubic], *F*(1,11) = 5.461, *P* = .039). There was no significant anterior–posterior variation but averaged across channel there were changes, particularly at the lower dose that differed between some high and low frequency bands (dose [linear] × frequency [order 4], *F*(1,11) = 8.037, *P* = .016; dose[quadratic] × frequency [order 4, 5], *F*(1,11) = 5.694, 5.71, *P* = .036, .036).

### Changes in Higuchi fractal dimension and frontal alpha asymmetry in MDD and PTSD

Analyzed together, including comorbid cases, there were no differences or interactions in HFD between TR-MDD and TR-PTSD (all *F*(1,31) < 1.2, all *P* > .25). There was a significant overall dose-related decrease in HFD with ketamine (dose [linear], *F*(1,31) = 4.843, *P* = .035, see Figure 3). There were no significant effects on frontal alpha asymmetry (FAA) (all *F*(1,31) < 3.45, *P* > .073, see Figure 3). With post-hoc analysis, neither TR-MDD nor TR-PTSD demonstrated the significant dose effect on HFD nor any effects on FAA.

### Changes in EEG frequency band power in OCD

**Figure 4:**
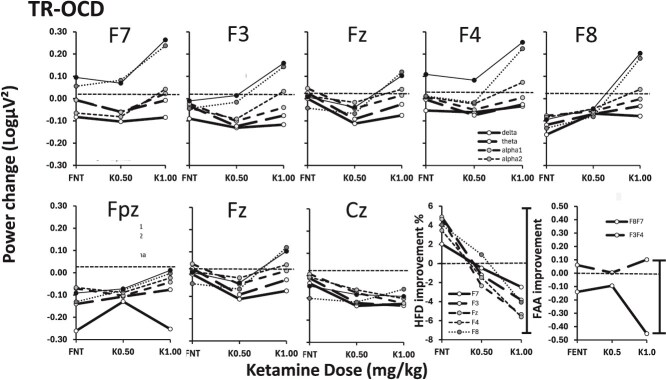
Upper and left panels: variation in post-pre power change by frequency band, channel, and ketamine dose for TR-OCD. Bottom right panels: Improvements in Higuchi’s fractal dimension (HFD%) across frontal channels and frontal alpha asymmetry for F8/F7 and F4/F3 electrode pairs averaged over α1 and α2 bands. Bars are the maximum of the individual data point SEs ×2 not of the trend components tested statistically

Separate analyses were carried out for left–right (F7 to F8) and anterior–posterior (Fpz–Cz) sets of channels. As can be seen in Figure 4, there is a lesser relative decrease with the intermediate dose of the bulk of low frequencies relative to the increase at the 2 higher frequencies at left compared to right channels (left–right, band [cubic] × dose [quadratic] × channel [linear], *F*(1,6) = 11.556, *P* = .015), but no such variation from Fpz to Cz (anterior–posterior, band [cubic] × dose [quadratic] × channel [linear/quadratic], *F*(1,6) <3.25, *P* > .2). Other apparent changes were not significant.

### Changes in HFD and FAA in OCD

F7 and F8 showed somewhat different HFD variation with dose than the midline channels (channel [quadratic] × dose [quadratic], *F*(1, 6) = 11.051, *P* = .016, Figure 4). With alpha asymmetry, F43α1 had negligible changes, while F87α2 showed a decrease with dose (dose [linear] × channel × band [linear], *F*(1, 7) = 6.971, *P* = .039).

### Correlation of changes in EEG frequency band power and clinical rating improvements

**Figure 5:**
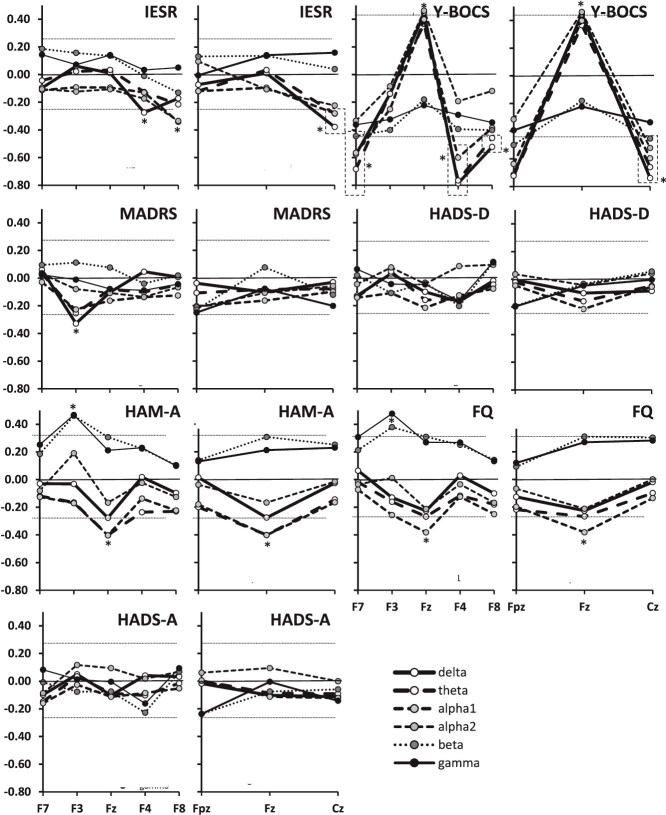
Correlations of 2-h post minus pre changes in power across different bands and channels versus changes in clinical scores between the same times. Left–right and anterior–posterior channel sets are plotted separately with Fz repeated. Clinical and power values were participant-centered to reflect drug-related but not participant-related changes. Statistical comparisons were corrected for false discovery rate over the whole pool of *P* values and significant values (at *P* < .05 after correction) are indicated by * with clusters of significant values in dashed boxes. Horizontal dashed lines show the critical value at *P* < .05 for **uncorrected** comparisons with the relevant sample sizes for each measure (note that Y-BOCS has the smallest N and so the largest critical value)

The correlations of the pre-post participant-centered clinical rating change and the pre-post participant-centered power change for each frequency band and channel are shown in Figure 5. A number of the correlations were found to be statistically significant after correcting for multiple comparisons using FDR, but the accuracy of the *value* of the correlations should be treated with caution as N is quite small (21-66), particularly in the case of Y-BOCS. For stability, *N* > 200 would be needed.^36^ These results are presented here primarily for qualitative comparison with each other and with Shadli, Kawe, Martin, McNaughton, Neehoff, and Glue^26^; and as a basis for the design of future experiments.

Patients assessed with IES-R were predominantly diagnosed with primary PTSD. The left–right variations in the correlation of EEG power change with IES-R change were complex and largely frequency-invariant with a significant decrease in delta at F4 and in alpha1 and alpha2 at F8. Rostro-caudal changes showed a trend to a caudal decrease at lower frequencies with significance for delta, theta, and alpha1 at Cz.

Patients assessed with Y-BOCS were predominantly diagnosed with primary OCD. The left–right variations in the correlation of EEG power change at lower frequencies (delta through alpha) with Y-BOCS change appear dramatic (likely as a result of small N) with significant negative correlations at F7, F4, and to some extent F8, and significant positive correlations at Fz. In contrast, the higher frequencies (beta and gamma) show weaker, generally negative correlations that are not significant. The anterior–posterior changes are very similar, with Fz showing opposite correlations to the other electrodes at the lower frequencies. The midline appears distinctive in both cases.

Patients diagnosed with MDD were all assessed with MADRS, HADS-D, and HADS-A. Bar a possible effect for MADRS at F3 in the delta range, there appear to be no effects - with a similar flat pattern across the measures and frequencies. HADS-A and HADS-D are very similar.

For patients with anxiety disorders as a secondary diagnosis (12 MDD, 2 PTSD), change in HAM-A had a negative relationship to power change at low frequencies that was greatest at Fz and significant for alpha 1 and alpha 2. It tended to have a positive relationship to changes at high frequencies (beta, gamma) peaking at F3. FQ results were essentially the same.

## Discussion

We found significant differences between MDD and PTSD in the dose-related changes produced by ketamine in the frequency band-power relationship, with post-hoc analysis finding qualitatively different patterns of results between them and between each and OCD. Overall (Figure 2), TR-MDD had little change with dose. TR-PTSD had an inverted-U-shaped effect of dose that was opposite in direction for the alpha2 and delta bands. In contrast, OCD had an increase in high frequencies at the high dose. The effects of the 0.5 mg/kg dose in the PTSD cases were clearest at the right frontal site, F8 (Figure 3), while the effects of the 1.0 mg/kg dose on beta and gamma in OCD were clearest at the left frontal site, F7 (Figure 4). These results contrast not only with each other but also with the previous results from GAD/SAD, where changes were most obvious at low frequency (delta, theta), high dose, and right-frontal sites.^26^

We also found significant relationships between power changes and changes in clinical scale scores. IESR change (pooled across all PTSD cases, ignoring comorbidities) shows a negative relationship to the 3 lowest bands (delta, theta, alpha1) at electrode Cz, to the 2 alpha bands at F8, and to delta at F4. As with the results of Shadli, Kawe, Martin, McNaughton, Neehoff, and Glue^26^ with theta at F4, there is no obvious basis for this specificity in the power change results. In contrast, Y-BOCS change has a negative relationship to the 3 lowest frequency bands at F4 and to a lesser extent at F8—again with no obvious basis for this specificity in the power curves. The F4 effect is consistent with the FQ correlation found by Shadli, Kawe, Martin, McNaughton, Neehoff, and Glue.^26^ Unlike the GAD/SAD results (where Fz is similar to but weaker than F4), there is an opposite, positive relationship of Y-BOCS to the same bands (and to alpha2). Neither MADRS nor HADS-D shows clear changes. MADRS may have an effect linked to delta at F3; this could be consistent with higher sensitivity than HADS-D.^37^ The qualitative pattern is similar across HAM-A and FQ, which differ from HADS-A. The theta change for HADS-A at Fz could be seen to be similar to the results of Shadli, Kawe, Martin, McNaughton, Neehoff, and Glue^26^ except that F4 is weaker, and they found their effect with FQ, which has a significant effect for alpha1 (not theta) at Fz (not F4). The positive relationship of both HAM-A and FQ to beta and gamma at F3 is unexpected.

The bulk of previous reports of the effects of ketamine on EEG and disorder have been limited to analysis of depression at doses ≤0.5 mg/kg and EEG testing times >3 h after administration (see Table 1).^38^ With early testing times, administration was consistently by low-dose intravenous infusion. This included a 45 min 0.25 mg/ kg infusion,^39^ a 40 min 0.5 or 0.2 mg/kg infusion (that was reported as producing headaches) with recording at 240 min post-infusion,^40^ a 0.25 mg/kg bolus followed by infusion at 0.25 mg/kg/h for 45 min,^41^ and 0.5 mg/kg infused over 40 min.^42^ While results are variable across studies, the overall tendency during or shortly after infusion is for ketamine to decrease power at low frequencies, eg, delta, and increase power at high frequencies, eg, gamma.^38^^,^^41^

Our analyses were exploratory with low N due to logistic constraints. It should also be noted that the bulk of patients continued on treatments established prior to study entry (Table 2), but the impact of any differences between these heterogenous treatments was not accounted for in the analysis. Note also that, at the time the study was devised, midazolam was commonly used as an active control but failed to adequately preserve study blinding. We, therefore, selected fentanyl at a dose commonly used for anesthetic pre-medication in the hope that this would be a safe and suitable control. The issue of a gold-standard ketamine control is still active, and we would not recommend that future studies use fentanyl as a control medication for ketamine.

Overall, while the precise patterns and specific values should be treated with caution, it seems safe to conclude that the effect of ketamine on resting EEG and the separate therapeutic links to resting EEG components (band and site) both vary with DSM diagnosis. This is particularly interesting in the case of the anxiety scales where, in contrast to Shadli, Kawe, Martin, McNaughton, Neehoff, and Glue,^26^ the anxiety reflects secondary comorbidity, not primary diagnosis. These results are consistent with the double-hit model of neurotic disorder (Figure 1). On this view, ketamine is altering a general neurotic component that determines sensitivity to aversion in general and is a necessary but not sufficient condition for disorder. The serotonin system is similar in providing a general background higher-order trait of “stability”^28^—but this is not specific to aversive events^29^ and much slower to alter disorder. In both cases, they indirectly change the impact of the high sensitivity of the disorder-specific systems on which conventional treatments act. The changes we have observed in the EEG, then, are likely to be the result of ketamine changing the pattern of neural activity specific to the disorder.

## Supplementary Material

MDD-PTSD-OCD_suppl_2025-06-30b_pyag037

## Data Availability

The EEG data and assigned diagnosis can be obtained from SMS sshadli@csu.edu.au or CKY calvin.young@otago.ac.nz on reasonable request. The clinical data are subject to privacy.
