# Supplementary material for: Ketamine effects on EEG and their links to therapy differ across treatment-resistant major depression, post-traumatic stress disorder, and obsessive-compulsive disorder
Source: Int J Neuropsychopharmacol. 2026 Jul 6;29(7):pyag037. doi: 10.1093/ijnp/pyag037 (PMC13425232; doi:10.1093/ijnp/pyag037)
Supplement: MDD-PTSD-OCD_suppl_2025-06-30b_pyag037 [file mdd-ptsd-ocd_suppl_2025-06-30b_pyag037.docx]

**Ketamine effects on EEG and links to therapy differ across treatment-resistant major depression, post-traumatic stress disorder, and obsessive-compulsive disorder**

**SUPPLEMENTARY MATERIAL**

Shabah M. Shadli^1,3^, Neda Nasrollahi^1^, Calvin K. Young^1^, Gabrielle S. R. Schuck^1^, Meadow G. Whatson^1^, Tame Kawe^1^, Shona Neehoff^2^, Ben Beagelhole^4^, Paul Glue^2^ and Neil McNaughton^1*^

**Affiliations:**

^1^Dept. Psychology, and ^2^Dept. Psychological Medicine, University of Otago, Dunedin, New Zealand. ^3^School of Psychology, Charles Sturt University, Bathurst, NSW, Australia. ^4^ Dept. Psychological Medicine, University of Otago, Christchurch, New Zealand. *Corresponding author.

**ORCID IDs:** SMS = 0000-0002-3607-3469; NN = 0000-0003-3923-4819; CKY = 0000-0002-6130-8370; GS = 0009-0000-8678-0893; BB = 0000-0002-9521-1745; TK = 0009-0006-9158-9536; PG = 0000-0002-7305-2800; NMcN = 0000-0003-4348-8221

**Correspondence to:** Professor Neil McNaughton

Department of Psychology

University of Otago

PO Box 56

Dunedin 9054

New Zealand

**Phone:** 64 3 479 7634; **Fax:** 64 3 479 8335; **Email**: [neil.mcnaughton@otago.ac.nz](mailto:neil.mcnaughton@otago.ac.nz)

**Words:** 2,150/4,000 (Exc. Abstract, References, Legends)

**Running title:** Ketamine, EEG, and Neurotic Disorders - Supplement

**Keywords:** ketamine**,** depression, post-traumatic stress disorder, obsessive-compulsive disorder, EEG

# Introduction

This supplement contains full (previously published) methods details.

# Supplementary Methods

*The following details are quoted with modifications from previous safety and efficacy reports on the same patients by Glue et al. (2024 for MDD), Beaglehole et al. (2025 for PTSD), and Beaglehole et al. (2024 for OCD).*

The protocol and consent forms for this study were approved by the Central Health and Disability Ethics Committee (19/CEN/21), and the study was registered with the Australian and New Zealand Clinical Trial Registry (ACTRN12619000311156). The protocol included recruitment of patients with treatment resistant MDD, PTSD, OCD, or spider phobia in separate cohorts, to evaluate the effects of ketamine on EEG biomarkers linked to trait neuroticism (McNaughton & Glue, 2020); only the TR-MDD cohort and TR-PTSD cohort are reported in this paper as primary data. This was a randomized double-blind psychoactive-controlled study in patients with treatment-resistant (TR, failure to respond to at least two conventional drugs and psychotherapy, Bokma et al., 2019) MDD or PTSD (DSM-5, American Psychiatric Association, 2013) and involved a direct comparison of the two. We also report preliminary significant results for OCD in a small sample (limited by recruitment and data loss), for comparison.

TR-D patients were interviewed using a structured clinical interview (Sheehan et al., 1998). Patient inclusion criteria included having a Montgomery-Åsberg Depression Rating Scale (MADRS, Montgomery & Asberg, 1979) score of ⩾20, being aged between 18 and 50 years, having good overall health and having had an inadequate response to prior treatment. Patients were permitted to have comorbid anxiety spectrum disorders. Exclusion criteria included evidence of severe or chronic medical disorders, past or current diagnoses of schizophrenia, bipolar disorder or current psychotic symptoms, current significant suicidal ideation, patients who were pregnant or lactating, substance use disorder or dependence in the last 6 months, and prior history of seizures or head injury. Patients provided signed informed consent before screening and were assessed as suitable to participate based on a review of medical history, safety laboratory tests (complete blood count, electrolytes, pregnancy test for patients who were capable of becoming pregnant), negative urine drug screening and vital signs. Patients were asked to provide a referral from a GP or psychiatrist who knew them well and could confirm the medical diagnosis and course of treatment. Patients were permitted to remain on current medication regimens and to continue with ongoing psychotherapy; however, no new treatments were to be started or changed.

TR-PTSD patients were interviewed using a structured clinical interview (Sheehan et al., 1998) by a psychiatrist (BB or PG), with treatment and data collection occurring in two locations (Dunedin and Christchurch, New Zealand). Patient inclusion criteria included having an Impact of Events Scale – Revised (IESR, Weiss & Marmar, 1997) score of >33, aged between 18-50 years, having good overall health and having had an inadequate response to prior antidepressant treatment. The study cohort was stratified into those with PTSD but no MDD; with a MADRS (Montgomery & Asberg, 1979) score of < 20 at screening), and patients with comorbid PTSD and TR-MDD (failure to respond to at least two antidepressants and psychotherapy and MADRS score of > 20 at screening) for sub-analysis. Exclusion criteria included evidence of severe or chronic medical disorders, past or current diagnoses of schizophrenia, bipolar disorder or current psychotic symptoms, current significant suicidal ideation, patients who were pregnant or lactating, patients with substance use disorder or dependence in the last 6 months, and prior history of seizures or head injury. The purpose of the exclusion criteria was to ensure that potential participants were stable and able to tolerate study participation in a psychiatric setting. Ethnicity (as per Stats NZ) was ascertained by self-report and from health records. Patients provided signed informed consent before screening, and were assessed as suitable to participate based on a review of medical history, safety laboratory tests (complete blood count, electrolytes, pregnancy test for patients who were capable of becoming pregnant), negative urine drug screening, and vital signs. Patients were asked to provide a referral from a GP or psychiatrist who knew them well and could confirm the medical diagnosis and prior treatment. Patients were permitted to remain on current medication regimens and to continue with ongoing psychotherapy, however, no new treatments were to be started or changed during the study.

OCD patients were recruited in two community settings (Dunedin and Christchurch, New Zealand, see Beaglehole et al., 2024 for CONSORT checklist). Participants were interviewed using a structured clinical interview (Sheehan et al., 1998). Inclusion criteria included having a Yale-Brown Obsessive Compulsive Scale (Y-BOCS, Goodman et al., 1989) score of >26, aged between 18-50 years, having good overall health, and having had an unsatisfactory response to at least two prior antidepressant treatments and at least one relevant psychotherapy. Included participants were required to not have a Montgomery Asberg Depression Rating Scale (MADRS, Montgomery & Asberg, 1979) score of >20 at screening. Other exclusion criteria included evidence of severe or chronic medical disorders, past or current diagnoses of schizophrenia, bipolar disorder or current psychotic symptoms, current significant suicidal ideation, patients who were pregnant or lactating, patients with substance use disorder or dependence in the last 6 months, and prior history of seizures or head injury. Ethnicity was ascertained by self-report and from health records. Participants provided signed informed consent before screening, and were assessed as suitable to participate based on a review of medical history, safety laboratory tests (complete blood count, electrolytes, pregnancy test for patients who were capable of becoming pregnant), negative urine drug screening, and vital signs. Participants were asked to provide a referral from a GP or psychiatrist who knew them well and could confirm the medical diagnosis and prior treatment. Participants were permitted to remain on current medication regimens and to continue with ongoing psychotherapy, however, no new treatments were to be started or changed during the study.

Study treatments included single doses of racemic ketamine 0.5 mg/kg, 1.0 mg/kg or fentanyl 50 mcg (psychoactive control). These were administered as intramuscular injections in the deltoid muscle, according to the technique proposed by the Health New Zealand | *Te Whatu Ora* (2024). Study drugs were given according to a computer-generated random code with balanced randomization, using a three-way within-subject double-blind active-controlled cross-over design. There were three dosing sessions, each session separated by at least 1 week. A 10-min relaxation EEG test was obtained pre-dose, and 2 h and 24 h after each dosing session to assess the timing of EEG changes in response to study treatments. Mood ratings and assessments of safety and tolerability were collected up to 168 h after each dose. Patients were monitored in the research clinic for a minimum of 2 h post-dose, with vital signs obtained pre-dose and at 15, 30, 45, 60, 90, and 120 min post-dose. Mood assessments for the MDD/PTSD patients included the MADRS (Montgomery & Asberg, 1979) pre-dose, and the Hospital Anxiety and Depression Scale (HADS, Zigmond & Snaith, 1983) pre-dose, at 60 and 120 min, and 24, 72 and 168 h post-dose. The choice of including the HADS scale as the main depression and anxiety rating scale was made after study registration but before final ethics committee approval and was to evaluate the use of a patient-rated outcome scale for these endpoints. Maintenance of blinding in participants and raters was not assessed.

Before patients were discharged from the research clinic, 2 h after dosing, we assessed their level of orientation, and recorded blood pressure and heart rate to check these were ⩽120% of baseline, that they were able to walk unassisted, were feeling physically well and not significantly sedated or distressed. If we had any concerns, we kept them in the clinic and reassessed them. Blinded safety data were reviewed during the study by an independent Data Safety Monitoring Board.

# References

American Psychiatric Association. (2013). *Diagnostic and statistical manual of mental disorders, fifth edition*. American Psychiatric Association.

Beaglehole, B., Glue, P., Neehoff, N., Shadli, S., McNaughton, N., Kimber, B., Muirhead, C., de Bie, A., Day-Brown, R., & Hughes-Medlicott, N. J. (2024). Ketamine for treatment-resistant Obsessive-Compulsive Disorder: double-blind active-controlled crossover study. *Journal of Psychopharmacology*, in press.

Beaglehole, B., Glue, P., Neehoff, S., Shadli, S., McNaughton, N., Kimber, B., Muirhead, C., de Bie, A., Day, R., & Hughes-Medlicott, N. J. (2025). Ketamine for treatment-resistant Post-Traumatic Stress Disorder: double-blind active-controlled randomised crossover study. Under revision.

Bokma, W. A., Wetzer, G., Gehrels, J. B., Penninx, B., Batelaan, N. M., & van Balkom, A. (2019). Aligning the many definitions of treatment resistance in anxiety disorders: A systematic review. *Depression and Anxiety*, *36*(9), 801-812. <https://doi.org/10.1002/da.22895>

Glue, P., Neehoff, S., Beaglehole, B., Shadli, S., McNaughton, N., & Hughes-Medlicott, N. J. (2024). Ketamine for treatment-resistant major depressive disorder: Double-blind active-controlled crossover study. *J Psychopharmacol*, *38*(2), 162-167. <https://doi.org/10.1177/02698811241227026>

Goodman, W. K., Price, L. H., Rasmussen, S. A., Mazure, C., Fleischmann, R. L., Hill, C. L., Heninger, G. R., & Charney, D. S. (1989). The Yale-Brown obsessive compulsive scale: I. Development, use, and reliability. *Archives of General Psychiatry*, *46*(11), 1006-1011.

Health New Zealand | *Te Whatu Ora*. (2024). *Immunisation Handbook, Version 5*. Retrieved from <https://www.tewhatuora.govt.nz/for-health-professionals/clinical-guidance/immunisation-handbook#pdf-download>

McNaughton, N., & Glue, P. (2020). Ketamine and neuroticism: a double-hit hypothesis of internalizing disorders. *Personality Neuroscience*, *3*, e2, Article e2. <https://doi.org/10.1017/pen.2020.2>

Montgomery, S. A., & Asberg, M. (1979). A new depression scale designed to be sensitive to change. *British Journal of Psychiatry*, *134*, 382-389.

Sheehan, D. V., Lecrubier, Y., Sheehan, K. H., Amorim, P., Janavs, J., Weiller, E., Hergueta, T., Baker, R., & Dunbar, G. C. (1998). The Mini-International Neuropsychiatric Interview (M.I.N.I): The development and validation of a structured diagnostic psychiatric interview for DSM-IV and ICD-10. *The Journal of clinical psychiatry*, *59*(Suppl 20), 22-33.

Weiss, D. S., & Marmar, C. R. (1997). The impact of event scale - revised. In J. P. Wilson & T. M. Kean (Eds.), *Assessing psychological trauma and PTSD* (pp. 399-411). Guilford Press.

Zigmond, A. S., & Snaith, R. P. (1983). The hospital anxiety and depression scale. *Acta Psychiatrica Scandinavica*, *67*(6), 361-370.
